# Aortic Arch Calcification as a Predictor of Repeated Arteriovenous Fistula Failure within 1-Year in Hemodialysis Patients

**DOI:** 10.1155/2017/6728437

**Published:** 2017-05-31

**Authors:** Yit-Sheung Yap, Kai-Ting Ting, Wen-Che Chi, Cheng-Hao Lin, Yi-Chun Liu, Po-Lin Kuo, Wan-Long Chuang

**Affiliations:** ^1^Graduate Institute of Clinical Medicine, College of Medicine, Kaohsiung Medical University, Kaohsiung 807, Taiwan; ^2^Division of Nephrology, Department of Internal Medicine, Yuan's General Hospital, Kaohsiung 802, Taiwan; ^3^Division of Gastroenterology, Department of Internal Medicine, Yuan's General Hospital, Kaohsiung 802, Taiwan; ^4^Division of Hepatobiliary, Department of Internal Medicine, Kaohsiung Medical University Hospital, Kaohsiung Medical University, Kaohsiung 807, Taiwan

## Abstract

**Objectives:**

The aim of the study was to identify the factors associated with repeated arteriovenous fistula (AVF) failure within 1-year, especially the impact of aortic arch calcification (AAC) on patency of AVF.

**Materials and Methods:**

We retrospectively assessed chest radiography in hemodialysis patients who had undergone initial AVF. The extent of AAC was categorized into four grades (0–3). The association between AAC grade, other clinical variables, and repeated failure of AVF was then analyzed by binary logistic regression analysis.

**Results:**

This study included 284 patients (158 males, mean age 61.7 ± 13.1 years). Patients with higher AAC grade were older, had more frequently diabetes mellitus and cardiovascular disease, had lower diastolic blood pressure, and had higher corrected calcium and lower intact parathyroid hormone levels. In multivariate analysis, the presence of higher AAC grade (odds ratio (95% confidence interval): 2.98 (1.43–6.23); *p* = 0.004), lower mean corrected calcium (*p* = 0.017), and mean serum albumin level (*p* = 0.008) were associated with repeated failure of AVF.

**Conclusions:**

The presence of higher AAC grade, lower mean corrected calcium and mean serum albumin level were independently associated with repeated AVF failure within 1 year in hemodialysis patients.

## 1. Introduction

Clinical practice guidelines recommend the arteriovenous fistula (AVF) as the preferred form of dialysis access owing to good long-term patency and a low incidence of complications [1]. Nonetheless, the AVF also has the disadvantages of longer maturation time and higher primary failure rate, which is usually caused by early thrombosis [2, 3]. A recent report pooled estimated primary AVF failure and 1-year primary patency to be 23% and 60% respectively [4]. Although percutaneous transluminal angioplasty (PTA) has generally replaced surgical procedures to be recognized as a standard approach to treat these stenotic lesions, its benefit is attenuated by a high re-stenotic rate within 6 months [1]. Of note, vascular access dysfunction is one of the leading causes of morbidity and mortality in hemodialysis patients and is also responsible for the high percentage of hospitalizations [5].

The main factors affecting primary patency loss of AVF are age, gender, the presence of cardiovascular disease, the preoperative diameter of the artery and vein, and so forth [3]. Besides, the major pathology cause in the development of AVF failure among hemodialysis patients is intimal hyperplastic stenosis [6]. Notably, vascular calcification including vascular access calcification and aortic arch calcification (AAC) are commonly seen in dialysis patients, and preexisting arterial calcification of the vascular access is also associated with worse AVF outcome in these populations [7–10]. Preexisting calcification may stiffen the artery and impair outward remodeling of the inflow artery, and thus limits its dilatation and resulting increase in blood flow after AVF operation [11]. A recent report even established that AAC, which is highly correlated with vascular access calcification, might predict primary patency loss of AVF [12]. Nevertheless, little has been published about whether repeated AVF failure is also affected by preexisting AAC.

The extent of AAC can be measured with electron beam computer tomography (EBCT) or multi-slice computer tomography (MSCT). However, these methods cannot be routinely performed due to the relatively high cost of examination and exposure to a high radiation dose [13, 14]. On the contrary, chest radiography is a non-invasive and inexpensive tool for the identification of AAC. Moreover, compared with plain hand film, chest radiography is routinely performed in all hemodialysis patients for cardiothoracic ratio assessment. Besides, the grading of vascular calcification of hand film could only be categorized into the presence of calcification or non-calcification [10]. Thus, chest radiography is recognized as a cost-effective and precise screening tool that can be used in place of plain hand film in examining vessel calcification in hemodialysis populations.

On the basis of these views, we hypothesized that the grading of AAC could be related to future development of repeated AVF failure. Therefore, the objective of this study was to identify factors predictive of repeated AVF failure within 1-year, especially the impact of AAC on AVF patency after an initial AVF creation among hemodialysis patients.

## 2. Methods

### 2.1. Ethics Statement

This study protocol was approved by the Institutional Review Board of Yuan's General Hospital, and the methods were performed in accordance with the Declaration of Helsinki. Requirement for patient consent was waived due to the minimal participant risk and retrospective nature of the study.

### 2.2. Study Population

All consecutive end-stage renal disease (ESRD) patients over 20 years of age who underwent first AVF surgery and started hemodialysis at dialysis centers of Yuan's general hospital between January 2006 and June 2015 were initially included in this retrospective observational study. Among these enrolled patients, subjects with follow-up duration of less than 1 year (*n* = 39), those who had no chest radiography records (*n* = 5), and those had received arteriovenous graft (AVG) recreation and permanent catheter placement within 1-year after AVF surgery (*n* = 7) were excluded. Finally, 284 patients were included in this study.

### 2.3. Clinical Variables

Clinical data including age, gender, comorbid diseases (diabetes mellitus, hypertension, and cardiovascular disease), body mass index (BMI), blood pressure (systolic and diastolic), medication, laboratory test and the AVF characteristics were extracted from medical records. Cardiovascular disease was defined as a history of coronary, cerebrovascular, or peripheral vascular disease: coronary disease was defined as a history of angioplasty, coronary artery bypass grafts, myocardial infarction, or typical angina and cerebrovascular disease as a history of cerebrovascular incidents such as cerebral bleeding or infarction, while peripheral vascular disease was defined as a history of prior revascularization procedure, amputation for ischemia or gangrene, or an ankle-brachial pressure index of <0.9. Diabetes was defined if fasting plasma glucose levels > 6.99 mmol/L, or glycated hemoglobin (HbA1c) > 6.5%, or if the patient was currently using hypoglycemic agents. Hypertension was defined if they had SBP ≥ 140 mm Hg, DBP ≥ 90 mmHg or a filled prescription for an antihypertensive medication. BMI was calculated as weight/height^2^ (kg/m^2^). Blood pressure was measured with an appropriate-sized cuff in the sitting position after a 5-min rest, using an automatic oscillometric monitor.

We evaluated baseline and mean laboratory tests including white blood cell, hemoglobin, uric acid, albumin, calcium, phosphorus, intact parathyroid hormone (IPTH), total cholesterol, and triglyceride. White blood cell and hemoglobin were measured on Beckman Coulter LH 750 (Miami, FL, U.S.A) using the electronic impedance method. Biochemical parameters (creatinine, uric acid, albumin, calcium, phosphorus, cholesterol, and triglycerides) were performed by standard laboratory procedure and measured on an Olympus AU2700 autoanalyzer (Beckman Coulter, Mishima, Japan) using an enzymatic assay. IPTH was assessed by a chemiluminescence assay. The mean laboratory levels were calculated from the time of the initial AVF occurrence to a follow-up period of 1 year. Meanwhile, calcium concentrations (mg/dL) were corrected for albumin concentrations (g/dL) using the following formula: corrected calcium = calcium + 0.8 × (4 − albumin).

All AVFs were created by three experienced surgeons (defined as A, B and C). With regard to our AVF placement strategy, normally our previous guideline was to start distally in the non-dominant arm and then proceed proximally and subsequently moving to the dominant arm for the purpose to preserve future vascular access options. However, the final decision regarding type and location of the initial access creation was individually based on clinical findings and surgeons' opinions.

### 2.4. Evaluation of Aortic Arch Calcification

**Figure 1:**
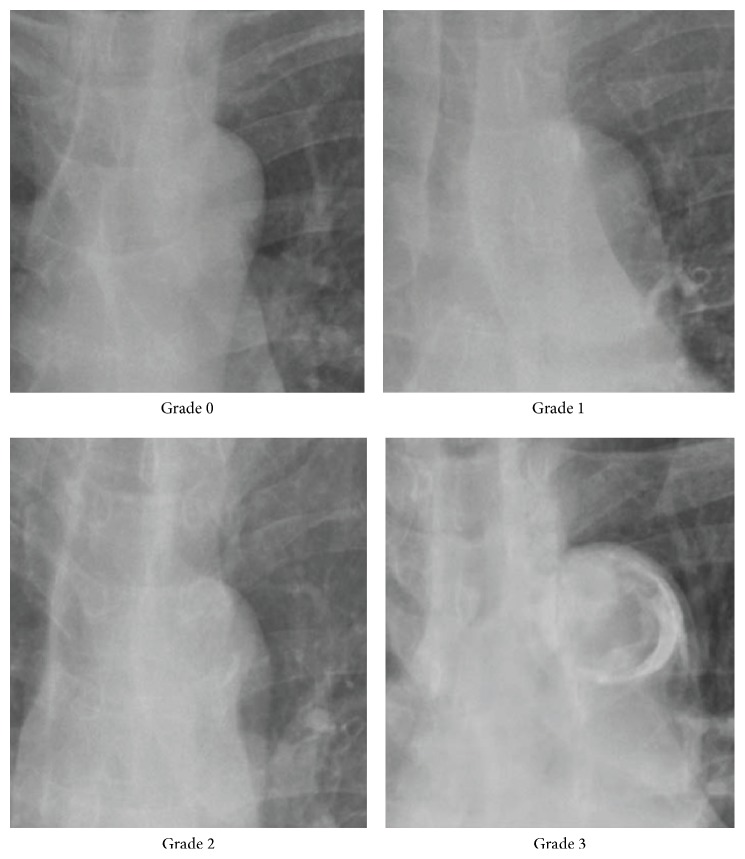
Representative images of aortic arch calcification on postero-anterior chest radiograph are shown. Aortic arch calcification extent is divided into four grades according to the categorization.

Two experienced medical doctors blinded to the patients' clinical data reviewed posterior-anterior plain chest X-rays taken at the time of AVF creation using a specific scoring system [15]. This scoring system, which divided the extent of calcification in the aortic arch into four grades, was as follows: grade 0, no visible calcification; grade 1, small spots of calcification or single thin calcification of the aortic knob; grade 2, one or more areas of thick calcification, but ≤50% of the circular area of the aortic knob; grade 3, circular calcification with >50% of circular area of the aortic knob. The extent of AAC in each chest radiography was evaluated as shown in Figure 1. Grades 0 to 1 and grades 2 to 3 were categorized as lower and higher AAC grade respectively. In all cases of disagreement between the physicians, consensus was eventually reached.

### 2.5. Clinical Outcome

AVF patency was followed up by physical examination, measurement of dynamic venous pressure, and measurements of access flow by the Transonic machine for 1 year postoperatively. AVF failure was defined as occurrence of thrombosis or requiring endovascular intervention (angioplasty or thrombectomy) to restore blood flow, and repeated AVF failure was defined as patients having at least two episodes of AVF patency loss within 1 year after surgery. As such, the conditions of failing AVF were defined as inadequate AVF blood flow (<600 mL/min), occlusion, elevated venous pressure (>150 mmHg or a trend of persistent increasing pressure over time), difficult cannulation and limited cannulation site, and other complications leading to nonfunctional access based on Kidney Disease Outcomes Quality Initiative (KDOQI) clinical practice guidelines [1].

### 2.6. Statistical Analysis

Statistical calculations were performed using the SPSS software program (SPSS Version 19; SPSS Inc., Chicago, IL, USA). Data were expressed as number with percentages for categorical values, and mean ± standard deviation for continuous variables. Medians and interquartile ranges were used for continuous variables without normal distribution. Chi-square test, independent* t*-test or Mann-Whitney test was used to analyze the differences in various variables between two groups of patients defined by AAC grade (lower grade versus higher grade).

We performed univariate and multivariate logistic regression analyses to determine associated factors of repeated AVF failure for all patients studied. Of this, all potential variables were included in the multivariate model (stepwise method). Test results were presented as odds ratio (OR) with 95% confidence intervals (CI), and two-sided *p* < 0.05 was considered statistically significant.

## 3. Results

### 3.1. Comparison of Clinical Characteristics between Lower Grade AAC and Higher Grade AAC

The study included 284 patients on regular hemodialysis with follow-up period of more than 1 year, 158 males and 126 females with mean age of 61.7 ± 13.1 years. A left-sided AVF was conducted in 206 (73.3%) patients, and a right-sided AVF was present in 75 (26.7%) patients. The forearm and upper arm for the AVF location was recorded in 244 (86.8%) and 37 (13.2%) cases respectively. Among these enrolled patients, 59 patients were categorized as Grade 0 (20.8%), 71 as Grade 1 (25.0%), 102 as Grade 2 (35.9%) and 52 as Grade 3 (18.3%).  Table 1 shows baseline data of patients between lower and higher AAC grades. The higher grade AAC group was older than the lower grade AAC group (66.5 ± 10.6 years versus 56.0 ± 13.6 years, *p* < 0.001), and had more cardiovascular disease (53.2% versus 29.2%, *p* < 0.001), diabetes (78.6% versus 66.9%, *p* = 0.027) and lower diastolic blood pressure (77.7 ± 11.8 mmHg versus 81.3 ± 13.9 mmHg, *p* = 0.019). In addition, compared to patients with lower grade, corrected calcium levels (2.17 ± 0.22 mmol/L versus 2.10 ± 0.25 mmol/L) were significantly higher, while IPTH (19.6 pmol/L versus 26.9 pmol/L) levels were significantly lower in the higher grade group. Other biochemical values (white blood cell count, hemoglobin, uric acid, phosphorus, albumin, total cholesterol and triglyceride) did not differ significantly between the two groups. On the other hand, there were also no significant differences in prevalence of sex and hypertension, BMI, AVF characteristics, and the use of medications between the two groups.

### 3.2. Risk Factors for Repeated AVF Failure within 1-Year

During the study period of 1 year, 54 (19.0%) patients experienced repeated AVF failure.  Table 2 shows the univariate logistic regression model for association of repeated AVF failure with all relevant factors. The univariate analysis identified higher grade AAC (OR (95% CI): 3.27 (1.66–6.42); *p* = 0.001), lower mean hemoglobin (*p* = 0.006), mean corrected calcium (*p* = 0.010) and mean serum albumin level (*p* < 0.001) to be significantly associated with repeated AVF failure. Then, we performed multivariate logistic regression analysis to identify the independent associated factors. As shown in Table 3, only the presence of higher grade AAC (2.98 (1.43–6.23); *p* = 0.004), lower mean corrected calcium (*p* = 0.017) and mean serum albumin level (*p* = 0.008) were associated with repeated AVF failure. Of note, the presence of comorbid conditions, AVF characteristics and surgeon factor were not associated significantly with repeated failure of AVF.

### 3.3. Summary of Repeated Failure of AVF according to AAC Grades

Table 4 demonstrates the summary of repeated failure of AVF. A total of 54 patients (19% of study patients) experienced repeated failure of AVF within 1 year, and all subjects were transferred for PTA. The total episodes of thrombosis or intervention in AAC grade 0, grade 1, grade 2 and grade 3 were 12, 15, 58 and 44 respectively. Besides, the repeated AVF failure rate at 1 year in each AAC grades was as follows: grade 0 (10.2%), grade 1 (9.9%), grade 2 (24.5%) and grade 3 (30.8%).

## 4. Discussion

The results of the present study established a high prevalence of AAC (79.2%) in hemodialysis patients, and 54.2% of them had higher grade calcification. Among these patients, 19.0% of them had repeated AVF failure within 1-year. Furthermore, we found that preexisting higher grade AAC identified in plain chest radiography, lower mean corrected calcium and mean serum albumin level were all independent risk factors for repeated AVF failure. To our knowledge, this is the first article to evaluate the role of AAC grade on repeated failure of AVF.

In this study, higher grade of AAC was linked to cardiovascular risk factors such as older age, diabetes mellitus and cardiovascular disease. These findings were consistent with results from previous reports [15, 16]. For this reason, these high-risk patients had increased rates of cardiac death as well as a higher overall mortality [17]. Otherwise, higher calcium and lower intact-PTH levels probably due to the decreased calcium-buffering capacity of bone were more prone to vascular calcification and thus associated with higher AAC grade [18]. Interestingly, patients with higher AAC grade had lower diastolic blood pressure. It was proposed that as the arteries became stiffer, the increased aortic pulse wave velocity (PWV) result in decreased diastolic blood pressure and high pulse pressure [19].

A previous report demonstrated that higher AAC grade predicted primary patency loss of AVF in ESRD patients [12]. The present study extends the previous finding that higher AAC grade is also a predictor of repeated AVF failure among hemodialysis populations. The reason of AAC-induced AVF failure is unclear, but we propose two possibilities. First, AAC could be a marker of systemic vascular calcification and arteriosclerosis. It has been shown that patients with AAC have linearly increased extent of calcification in the abdominal aorta and a trend toward more frequent arterial calcification of vascular access [20, 21]. Of note, AVF failure is significantly more common in patients with preexisting arterial calcification than in those without calcification [7, 8, 10]. Georgiadis et al. established that macro-calcification in the radial artery identified on a plain arm radiograph was correlated with a worse outcome of radiocephalic AVF in diabetic patients [10]. In another study reported by Choi et al., the result indicated that micro-calcification of artery determined by pathological examination of arterial tissue during AVF surgery was also associated with AVF failure within 1-year in incident hemodialysis patients [8]. In fact, vascular calcification and arterial stiffness might represent a stage of arteriosclerosis disease. The classification of AAC, in particular, has been shown to provide a significant role in estimating cardiovascular events with higher PWV (a simple way to measure arterial stiffness) corresponding with increasing elevations of risk. Importantly, the development of arteriosclerosis disease is not exclusive to the aortic arch, but rather is a systemic process that develops in multiple vessel beds including artery of dialysis access [17, 22]. As to our knowledge, it hinders the maturation process in AVF and subsequently contributes to access failure [23]. Second, AAC is linked to cardiovascular risk factors (such as older age, diabetes, etc.), which are also common predictor factors for AVF failure [3, 15, 16, 24]. As expected, patients with these high risk factors are easily prone to AVF dysfunction [3, 24]. Taken together, grading of AAC might indirectly indicate an increased risk of AVF failure and could serve as a noninvasive method for assessing overall AVF outcome in this population.

The issue regarding repeated failure of AVF remains one of the challenges for clinicians involved in vascular access care. Based on the findings of prior investigations, recurrent dysfunction of AVF within 6 months after initial PTA was seen in more than 25% of cases and most cases were recurrent stenosis [25]. Of note, AVF dysfunction is one of the leading causes of morbidity and mortality in hemodialysis patients and is responsible for their high percentage of hospitalizations [5]. Although the risk factors of repeated AVF failure particularly the factor of vascular calcification are rarely investigated, the relationship between vascular calcification and percutaneous intervention related-restenosis have been widely studied in cardiovascular diseases other than AVF [26]. In a pooled analysis of the HORIZONS and ACUITY trials that included 6855 subjects who underwent percutaneous coronary intervention (PCI), the presence of moderate/severe coronary calcification was a strong independent predictor of ischemic target lesion revascularization and 1-year definite stent thrombosis [26]. Likewise, stent expansion seemed to be inversely correlated with the arc of the calcium (*r* = −0.8; *p* < 0.001) [27]. In another recent report with patients undergoing cervical carotid artery stenting, the authors also identified calcified plaque as one of the risk factors to be significantly associated with the incidence of in-stent restenosis [28]. Additionally, among patients who underwent endovascular treatment for femoropopliteal disease, a significantly greater total volume of calcified plaque was found in patients developing restenosis (>50%) compared with those who did not, and they had a significantly worse amputation-free survival rate [29]. On the basis of these findings, severe vascular calcification is indeed a strong predictor of restenosis in the vast majority of cardiovascular diseases following endovascular treatment.

There are several potential mechanisms for vascular calcification induce-repeated failure in AVF. First, impaired vascular dilatation and outward remodeling induced by arteriosclerosis are suspected of being a cause of AVF maturation failure and restenosis. Notably, inadequate arterial and venous dilatation associated with lesion calcium might decrease AVF blood flow, and subsequently lead to restenosis of AVF [30]. Second, a correlation between the blood thrombogenicity and severity of calcification has been reported. A previous investigation established that thrombin level could predict the degree of coronary artery calcification, and suggested that thrombin plays a certain role in the pathophysiology of vascular calcification [31]. Therefore, it is proposed that AVF occlusion may occur under severe vessel calcification owing to thrombus formation. Third, vascular calcification is commonly accompanied by other pathological changes of the artery and vein such as intimal hyperplasia, medial fibrosis and venous calcification, which could synergistically contribute to AVF thrombosis [32, 33].

In our study, lower albumin and calcium level were related to the development of repeated AVF failure. It is known that both albumin and calcium as inflammatory factors impact on accelerated atherosclerosis and vascular calcification [34, 35]. In addition, chronic inflammation has also been suggested to be implicated in the pathogenesis and progression of intimal hyperplasia and subsequent AVF failure [36]. Of note, these markers were measured based on mean laboratory data instead of baseline level, which represented the mean effect of these factors on AVF outcome. However, to answer the question of causality with certainty, it would be required to evaluate whether restenosis could be attenuated by regimens that increase these serum levels. Hence, it remains elusive whether these molecules are simply a bystander or a contributor to restenosis.

Some limitations should also be considered in the interpretation of our study results. First, this semiquantitative method using four grades to assess AAC is relatively crude and may miss trivial calcium deposition in aortic arch. However, this condition would have no effect on result interpretation since this type of calcification would be categorized as lower AAC grade. Second, most study subjects were followed up mainly by clinical signs suggesting AVF failure; therefore, we may miss the possible presence of silent stenosis in some fistulas. Third, certain potential factors like circulating markers of inflammation, various calcification activators and inhibitors and vascular calcification of AVF were not measured in this study.

In summary, preexisting higher AAC grade, lower mean corrected calcium and mean serum albumin level were associated with repeated AVF failure within 1-year in hemodialysis patients. In fact, routine follow-up by chest radiography could be a simple and cost-effective tool to stratify AVF outcome in these patients. Besides, identification of these risk factors would allow the clinician to implement more rigorous monitoring and planned intervention for some risky new AVFs.

## Figures and Tables

**Table 1:** Comparison of baseline characteristics in the study patients with lower grade and higher grade aortic arch calcification.

Parameters	Total (*n* = 284)	Aortic arch calcification		*p *value
		Lower grade (*n* = 130)	Higher grade (*n* = 154)	
Age, years	61.7 ± 13.1	56.0 ± 13.6	66.5 ± 10.6	<0.001^*∗*^
Sex, *n* (%)				
Male	158 (55.6)	80 (61.5)	78 (50.6)	
Female	126 (44.4)	50 (38.5)	76 (49.4)	0.066
Cardiovascular disease, *n* (%)	120 (42.3)	38 (29.2)	82 (53.2)	<0.001^*∗*^
Diabetes mellitus, *n* (%)	208 (73.2)	87 (66.9)	121 (78.6)	<0.027^*∗*^
Hypertension, *n* (%)	276 (97.2)	124 (95.4)	152 (98.7)	0.092
Body mass index, kg/m^2^	24.9 ± 4.6	25.0 ± 4.9	24.8 ± 4.3	0.692
Systolic blood pressure (mmHg)	143.7 ± 22.0	144.5 ± 23.3	143.1 ± 21.0	0.581
Diastolic blood pressure (mmHg)	79.4 ± 12.9	81.3 ± 13.9	77.7 ± 11.8	0.019^*∗*^
Biochemical data (baseline value)				
White blood cell count, 10^9^/L	7.9 ± 3.2	7.8 ± 3.3	8.0 ± 3.0	0.549
Hemoglobin, mmol/L	1.35 ± 0.22	1.36 ± 0.22	1.35 ± 0.22	0.474
Uric acid, mmol/L	0.52 ± 0.15	0.51 ± 0.14	0.53 ± 0.16	0.111
Corrected calcium, mmol/L	2.14 ± 0.24	2.10 ± 0.25	2.17 ± 0.22	0.021^*∗*^
Phosphorus, mmol/L	2.00 ± 0.65	2.05 ± 0.68	1.95 ± 0.62	0.194
IPTH, pmol/L	23.0 (12.1–36.6)	26.9 (13.3–39.9)	19.6 (11.1–30.8)	0.021^*∗*^
Serum albumin, g/L	33.6 ± 5.8	33.8 ± 6.2	33.5 ± 5.4	0.674
Total cholesterol, mmol/L	4.47 ± 1.41	4.60 ± 1.44	4.36 ± 1.38	0.163
Triglyceride, mmol/L	1.78 ± 1.25	1.75 ± 1.35	1.81 ± 1.17	0.673
Medications				
Anti-platelet agent	110 (38.7)	48 (36.9)	62 (40.3)	0.565
ACEI/ARB	204 (71.8)	93 (71.5)	111 (72.1)	0.920
Lipid lowering agent	90 (31.7)	42 (32.3)	48 (31.2)	0.837
Site of AVF, *n* (%)				
Left side	206 (73.3)	94 (74.0)	112 (72.7)	
Right side	75 (26.7)	33 (26.0)	42 (27.3)	0.808
Location of AVF, *n* (%)				
Forearm	244 (86.8)	106 (83.5)	138 (89.6)	
Upper arm	37 (13.2)	21 (16.5)	16 (10.4)	0.129
Surgeon				
A	239 (84.2)	104 (80.0)	135 (87.7)	
B	18 (6.3)	11 (8.5)	7 (4.5)	
C	27 (9.5)	15 (11.5)	12 (7.8)	0.198

Abbreviations: IPTH = Intact parathyroid hormone; ACEI = Angiotensin converting enzyme inhibitor; ARB = Angiotensin receptor blocker; AVF = Arteriovenous fistula; Data are presented as mean ± standard deviation or numbers (percentages), except for IPTH, which are presented as median (interquartile range); ^*∗*^*p* < 0.05.

**Table 2:** Univariate analysis for the factors associated with the repeated arteriovenous fistula failure within 1 year after arteriovenous fistula creation.

Parameters	*N*	Comparison	OR (95% C.I.)	*p* value
Age	284	Per 1 year increase	1.01 (0.98–1.03)	0.617
Sex	158	Male	1	
	126	Female	1.45 (0.80–2.63)	0.220
Cardiovascular disease	164	No	1	
	120	Yes	1.02 (0.56–1.85)	0.955
Diabetes mellitus	76	No	1	
	208	Yes	1.35 (0.67–2.73)	0.404
Body mass index	279	Per 1 kg/m^2^ increase	1.00 (0.94–1.07)	0.989
Systolic blood pressure	281	Per 10 mmHg increase	0.98 (0.86–1.13)	0.797
Diastolic blood pressure	281	Per 10 mmHg increase	1.07 (0.85–1.34)	0.572
Biochemical data (mean value)				
White blood cell count	284	Per 10^9^/L increase	0.97 (0.86–1.10)	0.642
Hemoglobin	284	Per 0.16 mmol/L decrease	1.37 (1.10–1.72)	0.006^*∗*^
Uric acid	281	Per 0.059 mmol/L increase	1.11 (0.95–1.28)	0.187
Corrected calcium	284	Per 0.25 mmol/L decrease	1.69 (1.13–2.51)	0.010^*∗*^
Phosphorus	284	Per 0.323 mmol/L increase	1.16 (0.98–1.39)	0.089
IPTH	267	Per 10.6 pmol/L increase	0.925 (0.78–1.10)	0.377
Serum albumin	284	Per 10 g/L decrease	3.42 (1.88–6.25)	<0.001^*∗*^
Total cholesterol	281	Per 0.26 mmol/L increase	0.97 (0.90–1.04)	0.374
Triglyceride	283	Per 0.11 mmol/L increase	1.03 (1.00–1.06)	0.099
Medication				
Anti-platelet agent	174	No	1	
	110	Yes	0.92 (0.50–1.69)	0.776
ACEI/ARB	80	No	1	
	204	Yes	0.67 (0.35–1.25)	0.205
Lipid lowering agent	194	No	1	
	90	Yes	1.22 (0.65–2.27)	0.540

Site of AVF	206	Left	1	
	75	Right	1.07 (0.55–2.08)	0.841
Location of AVF	244	Forearm	1	
	37	Upper arm	0.98 (0.41–2.36)	0.961
Surgeon	239	A	1	
	18	B	0.78 (0.22–2.79)	0.697
	27	C	0.31 (0.07–1.36)	0.120
Aortic arch calcification	130	Lower grade	1	
	154	Higher grade	3.27 (1.66–6.42)	0.001^*∗*^

Abbreviations: *N* = Number of observations; OR = Odds ratio; C.I. = Confidence interval; IPTH = Intact parathyroid hormone; ACEI = Angiotensin converting enzyme inhibitor; ARB = Angiotensin receptor blocker; AVF = Arteriovenous fistula. Factor of hypertension was dropped out from analysis because of zero events in repeated arteriovenous fistula failure among patients without hypertension; ^*∗*^*p* < 0.05.

**Table 3:** Multivariate analysis (stepwise method) for the factors associated with the repeated arteriovenous fistula failure within 1 year after arteriovenous fistula creation.

Parameter	Comparison	OR (95%C.I.)	*p* value
Hemoglobin	Per 0.16 mmol/L decrease	—	0.147
Mean corrected calcium	Per 0.25 mmol/L decrease	1.77 (1.11–2.83)	0.017
Mean serum albumin	Per 10 g/L decrease	2.48 (1.26–4.88)	0.008
Aortic arch calcification	Lower grade	1	
	Higher grade	2.98 (1.43–6.23)	0.004

Abbreviations: OR = Odd ratio; C.I. = Confidence interval; Factor of hypertension was dropped out from analysis because of zero events in repeated arteriovenous fistula failure among patients without hypertension; Variables were dropped from the table if significant *p* > 0.05 except for hemoglobin.

**Table 4:** Summary of repeated failure of AVF divided in 4 aortic arch calcification grades.

	Aortic arch calcification grades			
	Grade 0	Grade 1	Grade 2	Grade 3
Total subjects, *n*	59	71	102	52
Subjects with repeated failure, *n*	6	7	25	16
Total episodes of thrombosis/intervention, *n*	12	15	58	44
Maximum episodes of thrombosis/intervention, *n*	2	3	4	5
Repeated failure rate at 1 year, %	10.2	9.9	24.5	30.8

Data are presented as numbers or percentages.

## References

[1] Vascular Access Work Group (2006). Clinical practice guidelines for vascular access. *American Journal of Kidney Diseases*.

[2] Lee T., Mokrzycki M., Moist L., Maya I., Vazquez M., Lok C. E. (2011). Standardized definitions for hemodialysis vascular access. *Seminars in Dialysis*.

[3] Lin C. C., Yang W. C. (2014). Clinical factors affecting patency of arteriovenous fistula in hemodialysis patients. *Acta Nephrologica*.

[4] Al-Jaishi A. A., Oliver M. J., Thomas S. M. (2014). Patency rates of the arteriovenous fistula for hemodialysis: a systematic review and meta-analysis. *American Journal of Kidney Diseases*.

[5] Schild A. F. (2010). Maintaining vascular access: the management of hemodialysis arteriovenous grafts. *Journal of Vascular Access*.

[6] Vazquez-Padron R. I., Allon M. (2016). New insights into dialysis vascular access: impact of preexisting arterial and venous pathology on AVF and AVG outcomes. *Clinical Journal of the American Society of Nephrology*.

[7] Jankovic A., Damjanovic T., Djuric Z. (2015). Impact of vascular calcifications on arteriovenous fistula survival in hemodialysis patients: a five-year follow-up. *Nephron*.

[8] Choi S. J., Yoon H. E., Kim Y. S. (2015). Pre-existing arterial micro-calcification predicts primary unassisted arteriovenous fistula failure in incident hemodialysis patients. *Seminars in Dialysis*.

[9] Jablonski K. L., Chonchol M. (2013). Vascular calcification in end-stage renal disease. *Hemodialysis International*.

[10] Georgiadis G. S., Georgakarakos E. I., Antoniou G. A. (2014). Correlation of pre-existing radial artery macrocalcifications with late patency of primary radiocephalic fistulas in diabetic hemodialysis patients. *Journal of Vascular Surgery*.

[11] Selvin E., Najjar S. S., Cornish T. C., Halushka M. K. (2010). A comprehensive histopathological evaluation of vascular medial fibrosis: insights into the pathophysiology of arterial stiffening. *Atherosclerosis*.

[12] Yap Y.-S., Ting K.-T., Chi W.-C., Lin C.-H., Liu Y.-C., Chuang W.-L. (2016). Aortic arch calcification predicts patency loss of arteriovenous fistula in end-stage renal disease patients. *Scientific Reports*.

[13] Raggi P. (2002). Effects of excess calcium load on the cardiovascular system measured with electron beam tomography in end-stage renal disease. *Nephrology Dialysis Transplantation*.

[14] Bellasi A., Raggi P. (2007). Techniques and technologies to assess vascular calcification. *Seminars in Dialysis*.

[15] Symeonidis G., Papanas N., Giannakis I. (2002). Gravity of aortic arch calcification as evaluated in adult Greek patients. *International Angiology*.

[16] Nitta K., Ogawa T. (2011). Aortic arch calcification and clinical outcome in patients with end-stage renal disease. *Tohoku Journal of Experimental Medicine*.

[17] Komatsu M., Okazaki M., Tsuchiya K., Kawaguchi H., Nitta K. (2014). Aortic arch calcification predicts cardiovascular and all-cause mortality in maintenance hemodialysis patients. *Kidney and Blood Pressure Research*.

[18] Kim S. C., Kim H. W., Oh S. W. (2011). Low iPTH can predict vascular and coronary calcifications in patients undergoing peritoneal dialysis. *Nephron - Clinical Practice*.

[19] London G. M., Guérin A. P., Marchais S. J., Métivier F., Pannier B., Adda H. (2003). Arterial media calcification in end-stage renal disease: impact on all-cause and cardiovascular mortality. *Nephrology Dialysis Transplantation*.

[20] Hashimoto H., Iijima K., Hashimoto M. (2009). Validity and usefulness of aortic arch calcification in chest X-ray. *Journal of Atherosclerosis and Thrombosis*.

[21] Kim H. G., Park S. C., Lee S. L. (2013). Arterial micro-calcification of vascular access is associated with aortic arch calcification and arterial stiffness in hemodialysis patients. *Seminars in Dialysis*.

[22] Tölle M., Reshetnik A., Schuchardt M., Höhne M., van der Giet M. (2015). Arteriosclerosis and vascular calcification: causes, clinical assessment and therapy. *European Journal of Clinical Investigation*.

[23] Bonucchi D., Cappelli G., Albertazzi A. (2002). Which is the preferred vascular access in diabetic patients? a view from Europe. *Nephrology Dialysis Transplantation*.

[24] Monroy-Cuadros M., Yilmaz S., Salazar-Bañuelos A., Doig C. (2010). Risk factors associated with patency loss of hemodialysis vascular access within 6 months. *Clinical Journal of the American Society of Nephrology*.

[25] Romann A., Beaulieu M. C., Rhéaume P., Clement J., Sidhu R., Kiaii M. (2016). Risk factors associated with arteriovenous fistula failure after first radiologic intervention. *Journal of Vascular Access*.

[26] Généreux P., Madhavan M. V., Mintz G. S. (2014). Ischemic outcomes after coronary intervention of calcified vessels in acute coronary syndromes: pooled analysis from the HORIZONS-AMI (harmonizing outcomes with revascularization and stents in acute myocardial infarction) and ACUITY (acute catheterization and urgent intervention triage strategy) trials. *Journal of the American College of Cardiology*.

[27] Vavuranakis M., Toutouzas K., Stefanadis C., Chrisohou C., Markou D., Toutouzas P. (2001). Stent deployment in calcified lesions: can we overcome calcific restraint with high-pressure balloon inflations?. *Catheterization and Cardiovascular Interventions*.

[28] Moon K., Albuquerque F. C., Levitt M. R., Ahmed A. S., Kalani M. Y., McDougall C. G. (2016). The myth of restenosis after carotid angioplasty and stenting. *Journal of Neurointerventional Surgery*.

[29] Patel S. D., Zymvragoudakis V., Sheehan L. (2015). Atherosclerotic plaque analysis: a pilot study to assess a novel tool to predict outcome following lower limb endovascular intervention. *European Journal of Vascular and Endovascular Surgery*.

[30] Asif A., Roy-Chaudhury P., Beathard G. A. (2006). Early arteriovenous fistula failure: a logical proposal for when and how to intervene. *Clinical Journal of the American Society of Nephrology: CJASN*.

[31] Borissoff J. I., Joosen I. A., Versteylen M. O., Spronk H. M., Ten Cate H., Hofstra L. (2012). Accelerated in vivo thrombin formation independently predicts the presence and severity of CT angiographic coronary atherosclerosis. *JACC: Cardiovascular Imaging*.

[32] Lee T., Chauhan V., Krishnamoorthy M. (2011). Severe venous neointimal hyperplasia prior to dialysis access surgery. *Nephrology Dialysis Transplantation*.

[33] Lee T., Safdar N., Mistry M. J. (2012). Preexisting venous calcification prior to dialysis vascular access surgery. *Seminars in Dialysis*.

[34] Turkmen K., Kayikcioglu H., Ozbek O. (2011). The relationship between epicardial adipose tissue and malnutrition, inflammation, atherosclerosis/calcification syndrome in ESRD patients. *Clinical Journal of the American Society of Nephrology*.

[35] Talmor-Barkan Y., Rashid G., Weintal I., Green J., Bernheim J., Benchetrit S. (2009). Low extracellular Ca2+: a mediator of endothelial inflammation. *Nephrology Dialysis Transplantation*.

[36] Wong C.-Y., De Vries M. R., Wang Y. (2014). Vascular remodeling and intimal hyperplasia in a novel murine model of arteriovenous fistula failure. *Journal of Vascular Surgery*.

